# Comparison Between Actigraphy Records and Parental Reports of Child's Sleep

**DOI:** 10.3389/fped.2020.567390

**Published:** 2020-09-24

**Authors:** Catarina Perpétuo, Marília Fernandes, Manuela Veríssimo

**Affiliations:** William James Center for Research, ISPA – Instituto Universitário, Lisbon, Portugal

**Keywords:** sleep, actigraphy, parental questionnaire, Bland and Altman method, preschoolers

## Abstract

Given the impact of sleep in several domains of a child's development, the comparison between actigraphy and parental questionnaires is of great importance in preschool-aged children, an understudied group. While parental reports tend to overestimate sleep duration, actigraphy boosts the frequency of night-waking's. Our primary goal was to compare actigraphy data and parental reports (*Children's Sleep Habits Questionnaire*, CSHQ), regarding bedtime, wake-up time, sleep duration, and wake after sleep onset (WASO), using the Bland–Altman technique. Forty-six children, age 3–6 years, and their parents participated. Results suggest that, despite existing associations between sleep schedule variables measured by both methods (from *r* = 0.57 regarding bedtime at weekends to *r* = 0.86 regarding wake-up time during the week, *ps*), differences between them were significant and agreements were weak, with parents overestimating bedtimes and wake-up times in relation to actigraphy. Differences between actigraphy and CSHQ were ± 52 min for weekly bedtime, ± 38 min for weekly wake-up time, ±159 min for total sleep time, and ± 62 min for WASO, indicating unsatisfactory agreement between methods. Correlations between actigraphy data and CSHQ dimensions are also explored. Our study contributes to the knowledge of the characteristics of each instrument, along with their tendency to overestimate and underestimate certain sleep parameters. We conclude that a complementary use of both instruments would better inform clinical practice and research on a child's sleep.

## Introduction

Sleep problems have implications in multiple areas of child development, for example in health conditions (1), behavioral problems (2), academic outcomes (3), and family conflict (4). Taking into account the high prevalence of sleep disturbance in childhood (20–30%) (5), we consider it fundamental to expand the knowledge about sleep measurement methods, as their variability limits comparisons between studies.

Polysomnography is based on neuropsychological parameters and is considered the most reliable objective sleep measurement method for determining sleep start/end times, as well as the frequency and length of night-walking's. However, it is an expensive and intrusive method that disrupts natural sleep patterns. Actigraphy is a validated alternative that provides continuous data collection in larger samples (6). It is a minimally invasive device that can be used over multiple nights in the child's natural environment, conferring ecological validity to collected data (7, 8). Nevertheless, it also presents some limitations (9) since sleep parameter estimation is based on monitoring activity. For example, the absence of movement that may occur during quiet activities can be registered as sleep periods (10), and movements during restless sleep episodes (typical of young children) (11) can be interpreted as night-waking's, impacting sleep parameter estimations (12–14). Nevertheless, objective sleep measures are consistently reported as more accurate than subjective ones (8, 11).

Subjective measures, such as sleep diaries or parental questionnaires, are widely used. They are simple and economic and allow access to environmental and behavioral dimensions related to bedtime routines that influence the child's sleep and cannot be measured with objective methods (15, 16). However, they are susceptible to response bias since they depend on what parents recall. Additionally, parents are sometimes not aware of their children's behaviors (e.g., night-waking's) as they depend on children's signaling (17). Sleep diaries are based on day-to-day descriptions (usually 7 days) of sleep parameters and are less likely to be biased by recall; however, this implies that parents use the diary regularly and rigorously.

Overall, there seems to be some convergence between actigraphy and parental reports for sleep schedule variables (e.g., sleep onset) but a lower or even a lack of convergence for sleep quality variables (e.g., night-waking's and sleep efficiency) (18).

Some studies using actigraphy alongside sleep diaries found significant associations between methods for bedtime and wake-up time and sleep duration, both in non-clinical samples (19, 20) and in samples of children diagnosed with severe nighttime fears (21). However, studies also reported that parents tend to overestimate sleep duration and wake-up time and to underestimate bedtime and night-waking's (22–26). On the one hand, parental reports tend to overestimate sleep duration, which may be explained by the child's increasing self-regulation capacity during night-waking's. On the other hand, actigraphy is vulnerable to over-detecting night-waking's in young children, whose sleep is typically more agitated (9). Different results are reported during a child's first year of life, when parents are more effective in reporting sleep schedule and duration variables, as well as occurrence and duration of night-waking's (27–29). Studies show more convergence between actigraphy and sleep diaries than with methods that use more global estimates (e.g., questionnaires) (18, 26, 29–31).

In a sample with older children (6–10 years old) using actigraphy and the *Children's Sleep Habits Questionnaire* (CSHQ) (32), significant associations were found between bedtime, time spent in bed, and sleep duration (33). Also, actigraphy-derived sleep duration was negatively associated with night-waking's and parasomnia dimensions, while objective sleep latency correlated with subjective reports of sleepiness (33).

Few studies have investigated sleep in preschoolers, which is surprising since in this phase sleep is very important to brain maturation, information processing, memory consolidation, and learning processes (34, 35). Even fewer studies have analyzed the concordance between different sleep measures. Most often they report correlations that do not provide appropriate information about agreement between methods.

The main aim of the current study is to evaluate relations between sleep parameters derived from actigraphy and CSHQ parental reporting for preschool-aged children. Specifically, we aim to (1) describe parental perceptions of children's sleep; (2) characterize sleep patterns on the basis of actigraphy; (3) relate CSHQ behavioral sleep dimensions with actigraphy data; and (4) report the agreement between actigraphy and parental reports according to Bland and Altman (36, 37).

## Methods

### Participants

We contacted 150 Portuguese families from the Lisbon metropolitan area and 54 agreed to participate in our study, conducted between January and October of 2019. Children were excluded from the sample if they had a neurological or psychological condition, or a diagnosed learning difficulty. Four families dropped out: three changed residence and one withdrew consent. The final sample included 46 participants (50% girls), with ages between 3 and 6 years (*M* = 4 years and 10 months, *SD* = 10.25 months); 21% had no siblings and 47% slept alone in their bedroom during the night. Children spent 7–10 h per day in daycare (*M* = 8.35, *SD* = 0.83).

Mothers' age ranged between 29 and 46 years (*M* = 38.10, *DP* = 4.41) and fathers' between 28 and 52 (*M* = 39.33, *DP* = 4.94). Mothers' education level ranged from 6 to 21 years (*M* = 15.14, *SD* = 3.53), and for fathers from 6 to 17 (*M* = 13.37, *SD* = 3.31). Mothers worked between 26 and 56 weekly hours (*M* = 38.32, *SD* = 5.35), and fathers worked between 35 and 60 hours per week (*M* = 40.37, *SD* = 5.60).

### Instruments

#### Actigraphy

All the children were asked to use the Actiwatch 2 (Philips Respironics, Murrysville, PA) on their non-dominant wrist, continuously for 7 days (M = 6.59, SD = 0.72). Actiwatch is a non-invasive accelerometer that collects data based on the child's motor activity, in the typical sleep environment. Retrieved data were coded into sleep and wake in 60-s (s) epochs using commercially available software (Actiware 6.0.9, Philips Respironics). Movements were scored using a default parameter of a medium wake threshold value of 40 counts per epoch (WTV-40). This level was chosen since night-waking is underestimated by high sensitivity and overestimated by low sensitivity. This software uses a validated algorithm to classify epochs as either sleep or wake: sleep onset was defined as the first period of 10 consecutive immobile minutes (min), and sleep offset as the last 10 consecutive immobile min, between bedtime and wake-up times. The software algorithm converted activity data in sleep estimates: (a) bedtime—the start time of the longest rest interval in that 24-h day; (b) wake-up time—the end time of the longest rest interval in the 24-h day; (c) time in bed—the sum of the durations for all rest intervals that are associated with the 24-h day; (d) total sleep time—the sum of the total sleep time for all sleep intervals associated with the 24-h day; (e) minutes onset latency—the sum of the onset latency for all sleep intervals associated with the 24-h day; (f) sleep efficiency—the total sleep time divided by time in bed and multiplied by 100; (g) wake after sleep onset (WASO)—the total number of minutes scored as wake within the sleep intervals associated with the 24-h day; and (h) number of night-waking—the total number of wake bouts within the sleep intervals associated with the 24-h day.

#### Parental Questionnaire

Children's Sleep Habits Questionnaire (CSHQ) (32, 38) was designed to evaluate behavioral dimensions and symptoms of sleep problems in children between the ages of 2 and 10. The psychometric properties of the instrument are satisfactory (32). The questionnaire has two types of questions: (a) quantitative—referring to bedtime and wake-up time (for weekdays and weekends), daily sleep time, and number and duration of night-wakings; (b) qualitative-−33 items distributed across 8 dimensions: *Bedtime resistance* (α = 0.75), *Sleep onset delay* (1 item), *Sleep duration* (α = 0.67), *Sleep anxiety* (α = 0.46), *Night-wakings* (α = 0.50), *Parasomnias* (α = 0.63), *Obstructive sleep apnea* (α = 0.51), and *Daytime sleepiness* (α = 0.51). It also includes a total score (α = 0.72). The *Sleep anxiety* dimension was excluded from the analyses due to low Cronbach's alpha. Items were answered on a 3-point Likert scale (1—rarely, 2—sometimes [2–4 times a week], 3—usually [5–7 times a week]). Higher scores indicate more disturbed sleep.

## Results

Before conducting the main analyses, we examined descriptive statistics for all the variables. We analyzed correlations and mean differences for parallel variables (same construct measured by both instruments), and the relation between CHSQ dimensions and actigraphy-measured sleep parameters. We also explored sex and age influences on sleep.

To evaluate the agreement between CSHQ and actigraphy, we used the Bland and Altman method (36, 37), a graphical approach that plots the differences between methods (i.e., CSHQ-Actigraph) with the mean methods (i.e., average [CSHQ, Actigraph]) and provides an interval where 95% of those differences are expected to lie (i.e., the limits of agreement). We defined satisfactory agreement as instances where these limits were <30 min (26).

### Descriptive Analyses of Parental Reported Data

Thirty-seven parents completed the CSHQ, and most of them (78.4%) reported that their children did not have sleep problems; only 8.1% stated sleep problems for their children. According to parents, children slept around 8–13 h per day. During the week, children went to bed between 9 and 11:30 _P.M._ and woke up between 6:45 and 9:30 _A.M._. On weekends, they went to bed significantly later [*t* (34) = −9.05, *p* < 0.001], between 9:30 _P.M._ and midnight, and also woke up significantly later, between 6:50 and 10:30 _A.M._ [*t* (35) = −6.79, *p* < 0.001]. Except for weekend wake-up time and night-waking length, no sex differences were found. Girls, compared to boys, woke up later on weekends [*t* (35) = 3.11, *p* < 0.01] and stayed wake less long during a night-waking [*t* (35) = −2.41, *p* < 0.05]. Finally, results showed that older children went to bed earlier on weekends (*r* = −0.43, *p* < 0.01), slept less (*r* = −0.38, *p* < 0.05), presented less bedtime resistance (*r* = −0.40, *p* < 0.01), woke up less at night (*r* = −0.39, *p* < 0.05), and presented globally fewer sleep problems (*r* = −0.33, *p* < 0.05). Table 1 presents means and standard deviations for parent-reported variables with respect to their child's sleep for the global sample, boys and girls.

**Table 1:** Means and standard deviations of parent-reported variables about a child's sleep from the CSHQ for the global sample, boys and girls.

		**Total**		**Boys**		**Girls**	
		**M**	**SD**	**M**	**SD**	**M**	**SD**
Bedtime (PM)	Week	9:51	00:34	9:55	00:36	9:48	0:32
	Weekend	10:31	00:38	10:24	0:35	10:37	0:39
Wake-up time (AM)	Week	7:52	0:35	7:47	0:37	7:57	0:34
	Weekend	8:46	0:56	8:20	0:48	9:12	0:52
Total sleep		10:28	1:06	10:24	1:16	10:31	0:59
Night-waking duration^*^		0:09	0:11	0:13	0:14	0:05	0:03
Bedtime resistance^*^		1.73	0.53	1.81	0.62	1.65	0.44
Sleep duration^*^		1.24	0.34	1.21	0.34	1.26	0.34
Night-waking's^*^		1.48	0.50	1.57	0.47	1.40	0.53
Parasomnias^*^		1.33	0.30	1.40	0.33	1.26	0.27
Obstructive sleep apnea^*^		1.11	0.26	1.18	0.36	1.05	0.12
Daytime sleepiness^*^		1.66	0.32	1.63	0.28	1.68	0.36
Global score		47.86	7.13	48.5	8.67	47.26	5.48

* *CSHQ's dimensional scores vary between 1 and 3, with higher scores corresponding to parental perception of higher frequency of sleep-related problems*.

### Descriptive Analyses of the Actigraphy Data

We obtained valid records for a total 41 children, who carried the Actiwatch between 5 and 9 days (*M* = 6.83, *SD* = 0.67). Actigraphy data are summarized in Table 2. Results showed that children slept between 6 h 15 min and 9 h 30 min per day. With respect to sleep schedules, during the week children went to bed between 9:30 and 12:15 _A.M._ and woke up between 6:45 and 9:15 _A.M_. During weekends, they went to bed significantly later, between 9:45 and 1:00 _A.M_ [*t* (41) = −4.24, *p* < 0.001], and woke up significantly later, between 7:00 and 10:00 _A.M_ [*t* (41) = −4.64, *p* < 0.001]. Results also showed that during the week girls, compared to boys, woke up significantly later [*t* (39) = 2.48, *p* < 0.05[ and spent more time in bed [*t* (39) = 2.10, *p* < 0.05]. A marginal sex effect was found on total sleep time, with girls sleeping more time than boys [*t* (39) = 1.97, *p* = 0.057]. A significant and negative correlation was found between time in bed and child's age (*r* = −0.41, *p* < 0.05). Time spent in daycare was significantly and negatively correlated with time in bed (*r* = −0.55, *p* < 0.001) and bedtime (*r* = 0.38, *p* < 0.05).

**Table 2:** Means and standard deviations for actigraphically recorded sleep parameters for the global sample, boys and girls.

		**Total**		**Boys**		**Girls**	
		**M**	**SD**	**M**	**SD**	**M**	**SD**
Bedtime	Week	10:25	00:35	10:25	00:36	10:25	00:36
	Weekend	10:52	00:49	10:47	00:46	10:56	00:51
Wake-up	Week	08:00	00:37	07:47	00:38	08:14	00:32
	Weekend	08:24	00:44	08:12	00:45	08:35	00:41
Time in bed		09:55	00:35	09:43	00:32	10:05	00:36
Total sleep		08:03	00:38	07:51	00:37	08:14	00:37
Sleep latency		00:13	00:16	00:11	00:08	00:15	00:21
Sleep efficiency		81%	6%	81%	6%	82%	7%
Wake after sleep onset (WASO)		01:22	00:28	01:22	00:23	01:21	00:33
Number of night waking's		35.16	6.81	36.85	6.96	33.70	6.48

### Associations Between Actigraphy and CSHQ Dimensions

Regarding the 34 children who used the Actiwatch and whose parents answered the CSHQ, we found that total sleep time from actigraphy was correlated with CSHQ *bedtime resistance* (*r* = −0.43, *p* < 0.05) and sleep problem *global score* (*r* = −0.38, *p* < 0.05). Actigraphy WASO was significantly and positively correlated with CSHQ *night-wakings* (*r* = 0.44, *p* < 0.05). More night-wakings registered by actigraphy were associated with parents' report of greater *instability on sleep duration* (*r* = 0.36, *p* < 0.05) and with more *parasomnias* (*r* = 0.35, *p* < 0.05). Higher actigraphy sleep efficiency was significantly and negatively correlated with parent reports on *bedtime resistance* (*r* = −0.42, *p* < 0.05), *night-wakings* (*r* = −0.45, *p* < 0.01), and *global scores* of sleep problems (*r* = −0.45, *p* < 0.01).

### Correlations, Differences, and Agreement Rates Between CSHQ and Actigraphy Parallel Variables

We found a positive significant correlation for bedtime, both during the week (*r* = 0.75, *p* < 0.001) and during the weekend (*r* = 0.57, *p* < 0.001), as well as for wake-up time (*r* = 0.86, *p* < 0.001 and *r* = 0.64, *p* < 0.001, during week and weekend, respectively). We did not find significant associations between methods for the sleep time nor for length of night-wakings (see Table 3).

**Table 3:** Associations between parallel variables recorded by both the actigraph and parental CSHQ reports.

	**Bedtime**		**Wake-up time**		**Total sleep**		**WASO**	
			**Week**	**Weekend**	**Week**	**Weekend**		
^**CSHQ**^	Bedtime	Week	0.75^**^	0.58^***^	0.53^**^	0.34	−0.22	−0.07
		Weekend	0.67^***^	0.57^***^	0.47^**^	0.43^*^	−0.13	0.03
	Wake-up	Week	0.49^**^	0.49^**^	0.86^***^	0.59^***^	0.12	0.14
		Weekend	0.39^*^	0.28	0.76^***^	0.64^***^	0.14	0.24
	Total sleep		0.10	−0.08	0.09	0.25	−**0.08**	0.33

* *p < 0.05*;

** *p < 0.01*;

*** *p < 0.001, bold values represent the correlations between same construct measured by both instruments*.

One-sample *t*-tests were conducted to analyze CSHQ and actigraphy differences. Parents reported earlier bedtimes than the actigraphy, both during the week [*t* (32) = −8.58, *p* < 0.001] and during weekends [t (30) = −2.37, *p* < 0.05], as well as earlier wake-up time during the week [*t* (33) = −2.18, *p* < 0.05]. Parents reported that children woke up later during weekends, compared with actigraphy [*t* (31) = 3.40, *p* < 0.01]. Finally, parents tended to overestimate total sleep time [t (31) = −10.55, *p* < 0.001] and to report shorter duration of night-wakings [*t* (32) = −13.75, *p* < 0.001] compared to the actigraphy results.

**Figure 1:**
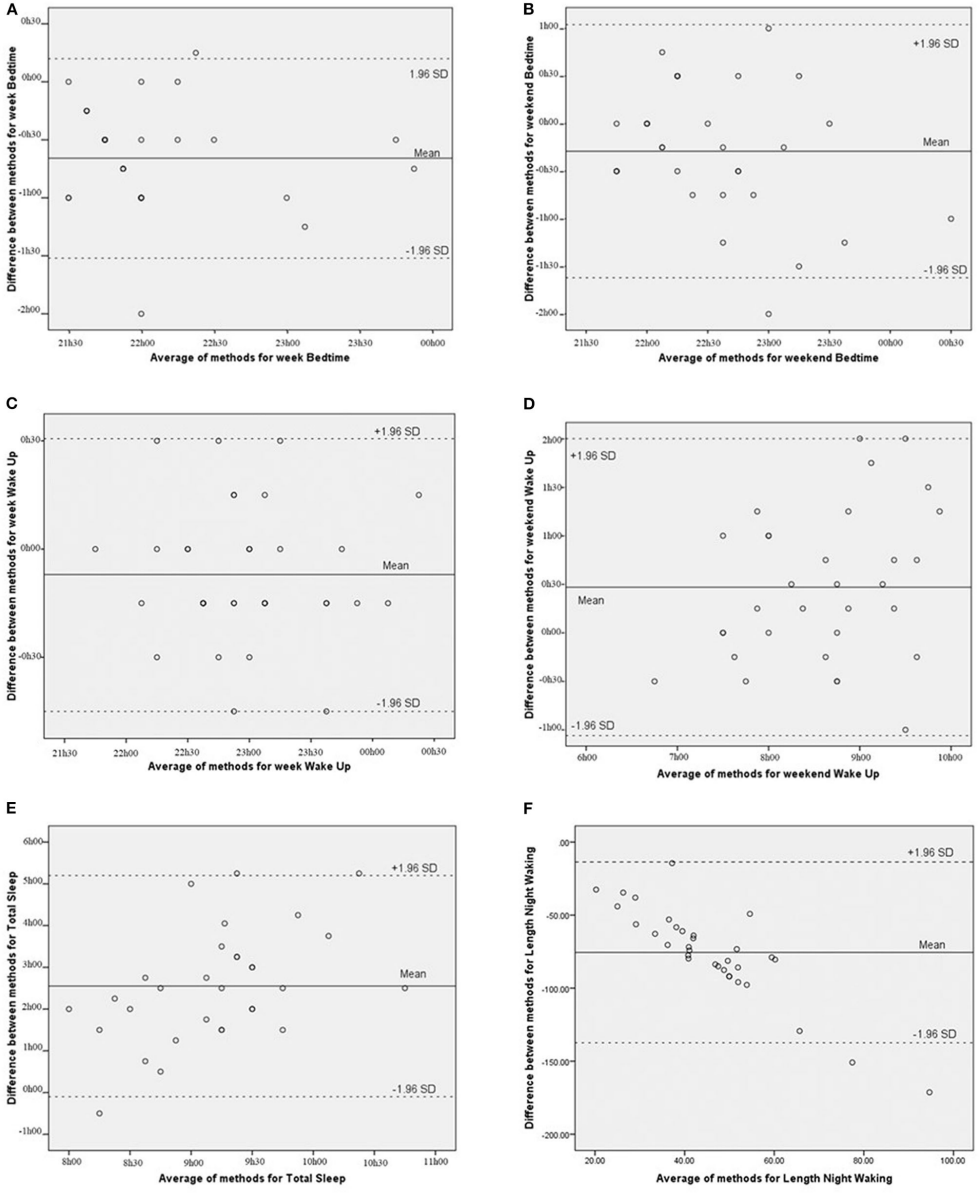
**(A-F)** Graphic representation of measurement differences between methods (CSHQ means - actigraph means) as a function of the mean differences between methods for specific parameters of bedtime (week and weekend), wake up time (week and weekend), total sleep time, and length of night waking.

In order to investigate the agreement between parent-reported and actigraphy values, we used the Bland and Altman method (36, 37). We calculated the mean differences for the data obtained by both methods (Mean [CSHQ – Actigraph]) and also the superior and inferior limits of agreement (± 1.96 × SD). Figure 1 plots the differences between the two methods (CSHQ-Actigraphy) with the mean of the methods (average [CSHQ; Actigraphy]) for each one of the considered parameters (Bedtime during week and weekend, Wake-up time during week and weekend, Total Sleep time, and Length of Night Waking). Based on previous studies (26), we defined 30 min as an acceptable difference between measures. With the exception of wake-up time during the week (see Table 3), there was no agreement between the parental reports and the actigraphy measures. During the week, parents reported a mean of 7 min earlier (*SD* = 0:19 min) wake-up time compared to time registered by actigraphy. Difference between methods is higher for weekends, where parents reported wake-up time 28 min (*SD* = 0:47) later than the actigraph's (see Table 4).

**Table 4:** Means of the differences between methods and agreement limits at 95%.

		**M**	**SD**	**Inferior limit**	**Superior limit**	**Interval**
Bedtime	Week	−0:40	0:26	−1:31	0:12	± 0:52
	Weekend	−0:17	0:41	−1:37	1:02	± 1:20
Wake-up	Week	−0:07	0:19	−0:45	0:31	± 0:38
	Weekend	0:28	0:47	−1:04	2:00	± 0:32
Total sleep		2:33	1:21	−0:06	5:12	± 2:39
WASO		−1:16	0:32	−2:17	−0:14	± 1:02

Concerning total sleep time and WASO, the longer the child slept (and the longer the night-wakings lasted) the greater the difference between methods was (β = 0.49, *p* < 0.01 and β = −0.88, *p* < 0.001 respectively). Therefore, there was a linear relation between average values and between-method differences, which represents a serious threat to a parametric Bland and Altman analysis. For this reason, we used the non-parametric approach of the Bland and Altman method (36, 37). Results showed that, for total sleep time, only 6.5% of the pairs of observations showed a difference of 30 min or less, and for 25.8% of the pairs of observations, the difference was of 1 h 30 or less. For WASO, only for 3% of the paired observations was the difference < 30 min; for 27%, the difference was < 1 h. For all other variables, the difference was > 1 h.

## Discussion

The present study aimed to explore sleep patterns in preschool children and to compare results derived from objective (i.e., actigraphy) and subjective (i.e., CSHQ) methods. Descriptive results were in line with previous literature: children went to bed and woke up later on weekends than on school days (39–41), which might be linked to stricter impositions from school-related wake-up times and more flexible routines during weekends. Although children in our sample go to bed and wake up later than reported on a meta-analysis by Galland et al., our sample's mean sleep efficiency of 81% lays on the interval found by those authors for children between the age of 3 and 14 years (42).

According to the parents, older children presented shorter sleep duration, less bedtime resistance, fewer night-wakings, and globally less sleep problems. Actigraphy results showed that they spent less time in bed. During the preschool years, sleep patterns were more organized, stable, and less disrupted, due to maturational reasons (33, 43–45).

Children who slept more according to actigraphy were described by the parents as having less bedtime-resistant behaviors. Equivalent findings were obtained by Holley et al. (33) with older children. On the one hand, a child who sleeps fewer hours could experience higher levels of sleepiness during the day, becoming more irritable, and this could be reflected in reluctance to go to bed at night (46). On the other hand, a child who resists going to bed may delay sleep start time, which translates to less total sleep amount (47).

Actigraphy results for total sleep time and sleep efficiency were also associated with parents reporting globally less sleep problems, suggesting that these parameters might be important when considering sleep problems. Some studies assert that inadequate sleep quantity and quality reflect the existence of sleep problems (32, 48, 49). Sleep efficiency was also correlated with less *bedtime resistance*, which may mean that a child who peacefully accepts bedtime has fewer difficulties initiating and/or maintaining sleep, spending asleep most of the time in bed. Finally, sleep efficiency was associated with fewer behavioral manifestations of *night-wakings*. We could not establish causality in this relation; however, it is theoretically plausible that night-wakings perceived by parents (corresponding to periods the child spends asleep during night-time) indicated decreased sleep efficiency.

Although objectively and subjectively measured minutes of *wake after sleep onset* (WASO) were different, we found a significant association between the first one and the *night-waking* CSHQ dimension, meaning that the longer a child was awake after sleep onset, the more the parents tended to report night-wakings. It is possible that as the child spends more time awake during the night, he will be more likely to request parental intervention. Higher number of night-wakings measured by actigraphy was associated with parental reports of more *sleep duration instability* and also of *parasomnias*, which suggests that behavioral aspects linked to this kind of sleep disruption can translate into increased motor activity during night, raising the number of actigraphy night-wakings (33).

Consistent with previous findings (25), despite significant correlations between questionnaires and actigraphy for sleep schedule variables, differences were also significant, with parents reporting consistently earlier bedtimes and later wake-up times than actigraphy (23, 50, 51). This may be due do parents assuming that the child falls asleep as soon as he/she goes to bed, not considering the time it takes for him/her to fall asleep.

We also found significant differences between measures for sleep duration and WASO. Parental overestimation of sleep duration reflected the occurrence of unnoticed night-wakings and consistent reports of earlier bedtimes and later wake-up times compared with actigraphy (20, 23). Actigraphy results on WASO were significantly higher than parental reports, which is in line with other studies (26, 27, 33, 52), even for children in their 1st year (29). This discrepancy may be due either to a tendency for actigraphy to mistakenly overestimate night-wakings when the child's sleep is restless (9) or to a parental inability to report them, as the child's growing self-regulation tools allow him to resume sleep without requesting parental intervention. Additionally, given that our participants came from a population without diagnosed sleep problems and their parents tended to evaluate their sleep quality as good, parents may not have felt the need to monitor night-wakings so closely. This hypothesis is strengthened by Kushnir and Sadeh (22), who proposed that the existence of diagnosed sleep problems makes parents more vigilant and sensitive when monitoring and reporting child's sleep than parents of good sleepers.

Regarding the agreement between both methods, we did not find satisfactory agreement rates for any of the considered variables (36, 37), with the exception of wake-up times during week (near the 30 min reference) (26). The few studies evaluating agreement between parallel variables measured by actigraphy and parental reports presented mixed findings (26, 28, 29, 53). Tikotzky and Volkovich (29) obtained satisfactory agreement between actigraphy and questionnaires for children until they were 18 months old. Werner et al. (26) attained satisfactory agreement intervals for bedtimes and wake-up times, but not for sleep duration and night-wakings, while Bélanger et al. (53), in line with our results, did not obtain concordance for any of the measured patterns. Actigraphy recorded motor activity data continuously for 7 days and the questionnaire elicited global parental perceptions about the same 7 days. On the one hand, we know that parental answers are frequently vague and imprecise (26), even more so if compared with objective measures. On the other hand, actigraphy records phenomena outside of parental awareness, such as during night periods.

Overall, our results suggest there might be an advantage to using parental reports and actigraphy complementarily. Although parents can provide relevant reports of behavioral dimensions surrounding the child's sleep, actigraphy can overcome gaps in parental perceptions estimating sleep parameters, which are dependent on the child signaling nocturnal sleep-related events. This is especially relevant at the preschool age, when children become less dependent on parents for resuming sleep. Exploring to what extent these methods are discrepant from one another might be useful to better understand children's sleep patterns and contexts, as well as being a good start for identifying possible sleep problems.

### Limitations and Future Directions

Our relatively small-sized sample was recruited from middle-class populations, which limits the interpretation of our results. Another limitation is the lack of sleep diary data. The measures used in the present study invite parents to formulate a generic retrospective answer about sleep schedules, duration, and night-waking while the sleep diary registers the child's daily sleep. We suggest that in future studies sleep questionnaires and diaries should be used simultaneously to record child sleep.

Despite these limitations, our study is notable for being the first to evaluate associations between objective sleep parameters and behavioral sleep dimensions associated with sleep habits and routines in preschool-aged children. Making sense of these associations constitutes an important improvement in the understanding of the contributions of both methods of measuring child's sleep, in both clinical and research contexts, given its importance in different domains of cognitive and social child development.

## Data Availability Statement

The raw data supporting the conclusions of this article will be made available by the authors, without undue reservation.

## Ethics Statement

The studies involving human participants were reviewed and approved by Ethics Commission of ISPA's Center for Research. Written informed consent to participate in this study was provided by the participants' legal guardian/next of kin.

## Author Contributions

CP collected, coded the data, and drafted the manuscript. MV planned the study. CP, MF, and MV interpreted the data, gave final approval for the version to be published, and agreed to be accountable for all aspects of work. All authors contributed to the article and approved the submitted version.

## Conflict of Interest

The authors declare that the research was conducted in the absence of any commercial or financial relationships that could be construed as a potential conflict of interest.
